# Identifying Early Mild Cognitive Impairment by Multi-Modality MRI-Based Deep Learning

**DOI:** 10.3389/fnagi.2020.00206

**Published:** 2020-09-04

**Authors:** Li Kang, Jingwan Jiang, Jianjun Huang, Tijiang Zhang

**Affiliations:** ^1^College of Information Engineering, Shenzhen University, Shenzhen, China; ^2^Department of Radiology, The Affiliated Hospital of Zunyi Medical University, Zunyi, China

**Keywords:** early mild cognitive impairment, multi-modality diagnosis, convolutional neural network, support vector machine, transfer learning

## Abstract

Mild cognitive impairment (MCI) is a clinical state with a high risk of conversion to Alzheimer's Disease (AD). Since there is no effective treatment for AD, it is extremely important to diagnose MCI as early as possible, as this makes it possible to delay its progression toward AD. However, it's challenging to identify early MCI (EMCI) because there are only mild changes in the brain structures of patients compared with a normal control (NC). To extract remarkable features for these mild changes, in this paper, a multi-modality diagnosis approach based on deep learning is presented. Firstly, we propose to use structure MRI and diffusion tensor imaging (DTI) images as the multi-modality data to identify EMCI. Then, a convolutional neural network based on transfer learning technique is developed to extract features of the multi-modality data, where an L1-norm is introduced to reduce the feature dimensionality and retrieve essential features for the identification. At last, the classifier produces 94.2% accuracy for EMCI vs. NC on an ADNI dataset. Experimental results show that multi-modality data can provide more useful information to distinguish EMCI from NC compared with single modality data, and the proposed method can improve classification performance, which is beneficial to early intervention of AD. In addition, it is found that DTI image can act as an important biomarker for EMCI from the point of view of a clinical diagnosis.

## 1. Introduction

Alzheimer's disease (AD) is an irreversible degenerative brain disease and the most common cause of dementia (Wilson et al., 2012), which often happens to people aged over 65 years. In Alzheimer's disease, neurons in parts of the brain involved in cognitive function are eventually damaged or destroyed. In 2018, the estimated number of AD patients was 5.7 million in America, and this number will continue to grow to 13.8 million by 2050 (Alzheimer's Association, 2018). There is no effective prevention or treatment against AD at present. Early Mild Cognitive Impairment (EMCI) is the stage between age-related cognitive decline and AD or other types of dementia (Schneider, 2006). Early intervention against EMCI will possibly delay the progression of EMCI toward AD.

People with MCI exhibit mild but measurable changes in thinking abilities. A systematic review found that an average of 32% individuals with MCI developed into AD within 5 years, which shows that MCI patients have a high risk of conversion to AD (Alzheimer's Association, 2018). Early diagnosis of MCI is therefore of great importance to early intervention in the preclinical state of AD (Lim, 2016; Wen and Li, 2019), and this has received extensive attention from researchers in the recent decades (Jiang et al., 2020). However, identifying EMCI is a challenging clinical problem due to the mild changes between EMCI and NC.

Many studies utilize machine learning methods to complete computer-aided diagnosis for EMCI in which various neural imaging techniques, such as structure Magnetic Resonance Imaging (sMRI), functional MRI (fMRI), and diffusion MRI (dMRI), work as data sources. Based on dMRI [specifically, Diffusion Weighted Imaging (DWI)], Prasad et al. (2015) calculated a 68 × 68 connectivity matrix and a set of network measures from 68 cortical areas as the input of support vector machine (SVM), and a classification accuracy of 59.2% was achieved for EMCI vs. NC. Using sMRI data, Raeper et al. (2018) proposed a cooperative correlational and discriminative ensemble learning framework. Each individual brain was represented by a set of shallow convolutional brain multiplexes (SCBMs) used to train an ensemble of canonical correlation analysis (CCA)-SVM and linear discriminative analysis (LDA)-based classifiers, achieving an accuracy of 80.95%. Kang and Suk (2018) separated the fMRI signals into a true source signal and a noise component by means of a stochastic variational Bayesian inference and then calculated source correlations of the inferred source signals as input features of a linear SVM, achieving an accuracy of 74.45%. Chen et al. (2016) and Jiao et al. (2019) also utilized fMRI data, they constructed low-order and high-order functional networks to train SVM and achieved an accuracy of 88.14 and 91.13%, respectively. It worth noting that Jiao et al. performed least the absolute shrinkage and selection operator (LASSO) feature selection algorithm.

The above methods based on tradition machine learning commonly need complex feature engineering to extract region of interest (ROI)-based or voxel-wise features used for a classification task. The validity of the extracted features thus largely depends on image preprocessing steps, such as segmentation and registration, as well as prior hypotheses. Recently, researchers have shown an increasing interest in the convolutional neural network (CNN) in medical image classification field (Islam and Zhang, 2017; Yue et al., 2019). The CNN can alleviate the above problems by automatically extracting the most discriminating disease-related features from voxel values of complex high-dimensional image data in end-to-end modes, which is conducive to avoiding errors caused by feature engineering and retains the subtle differences between EMCI and NC.

Some studies have tried to use the CNN to extract latent features of neuroimaging data for EMCI classification. Kam et al. (2019) proposed a novel 3DCNN framework to extract deeply embedded features from both static and dynamic brain functional networks of fMRI data for EMCI classification, and they reported an accuracy of 76.07%. However, time-consuming, multi-channel, and multi-model training did not result in higher classification accuracy. Yue et al. (2018) utilized a 2DCNN to acquire the most useful features of the gray matter of sMRI. This deep learning method achieved high accuracy for EMCI vs. AD and LMCI vs. EMCI, but the classification task excluded EMCI vs. NC. Puranik et al. (2018) employed a 2DCNN model with transfer learning technique to classify AD, EMCI, and NC and obtained an accuracy of 98.41%. However, the inputs of the CNN are the 2D slices of fMRI images, which means that the classification task is not based on subject-level, deviating clinical needs. Apart from one paper, the above methods did not process the problem of binary classification of EMCI vs. NC (Kam et al., 2019).

On account of the similar brain structure and brain functions between EMCI and NC, it is very challenging to distinguish EMCI from NC. The diagnostic accuracy of EMCI in all the above studies is much lower than that of AD or MCI due to the subtle differences between EMCI and NC. On the other hand, with single modality data it is difficult to extract enough features to classify EMCI from NC (Qi et al., 2014; Cabrera-León et al., 2018). For example, sMRI data cannot catch the mild changes of brain structure. Since multi-modality data can provide more useful auxiliary information, it seems to be more promising to extract the most discriminative features to perform the classification task.

Fortunately, multi-modality-based diagnosis has attracted extensive attention and become a hot area of research within medical image analysis. Some studies have successfully applied multi-modality neuroimaging analysis to the diagnosis of AD, as can be seen in Baiying et al. (2016), Khvostikov et al. (2018), and Cheng and Liu (2017). For EMCI classification using multi-modality data, Forouzannezhad et al. (2018b) made a preliminary attempt. They combined the features extracted from cortical region and subcortical region of sMRI and PET images to train a deep neural network (DNN) and achieved an accuracy of 84%. The authors also trained a SVM classifier utilizing the same data in paper (Forouzannezhad et al., 2018b) and reported an accuracy of 81.1% (Forouzannezhad et al., 2018a).

In this paper, we further develop a multi-modality diagnosis method for EMCI. Specifically, we fused sMRI and DTI data with the multi-modality fusion strategy and then combined the CNN model and SVM classifier to identify EMCI. To the best of our knowledge, we are the first to utilize DTI data to train CNN for EMCI diagnosis. DTI data has been proven to be a useful diagnostic marker for distinguishing EMCI from NC, especially its measures, namely fractional anisotropy (FA) and mean diffusivity (MD). Moreover, DTI data can reflect brain microstructure changes by quantifying the integrity of white matter (Nowrangi et al., 2013; Marizzoni et al., 2016; Brueggen et al., 2017; Gyula, 2018), which makes it promising for identifying the subtle differences of EMCI compared with NC.

## 2. Materials and Methods

### 2.1. Data Acquisition

sMRI and DTI data used in this study were obtained from Alzheimer's Disease Neuroimaging Initiative (ADNI) project, which was launched in 2003 as a public-private partnership. The goals of the ADNI study are to identify biomarkers for clinical use and detect AD at the early stage (EMCI, MCI, and LMCI) using biomarkers. Multiple biomarkers, including sMRI, fMRI, DTI, and related neuropsychological assessments, are combined in an effort to detect the progression of EMCI and early AD.

In this study, we selected 120 subjects, including 70 EMCI subjects and 50 age-matched Normal Controls (NCs) from the ADNIGO and ADNI2 database. For each subject, there is a T1-weighted sMRI image, a FA-DTI image, and an MD-DTI image in an NIfTI file format. The selected subjects coming from the basebline/screening visit have passed strict inclusion criteria. Table 1 shows the detailed demographic information of subjects used in this study, where Mini Mental State Examination (MMSE) is a mental test quantifying the cognitive function. As an auxiliary diagnostic index, the lower MMSE score indicates poor cognitive ability.

**Table 1:** Corresponding statistical information of subjects.

	**EMCI**	**NC**
Number	70	50
Gender(F/M)	27/43	27/23
Age(year)	72.9 ± 8.3	72.5 ± 6.1
MMSE	27.86 ± 1.66	28.93 ± 1.18

All raw data were acquired by 3T GE medical system scanners at multiple sites with a rigorous quality control to reduce site effect. The raw 3D T1-weighted sMRI scans were collected with the following imaging parameters: 256 × 256 × 196 voxels per volume, 1.0 × 1.0 × 1.2 *mm*^3^ every voxel size, inversion time = 400 ms, and flip angle = 11°. The raw DTI data of each subject is formed of 41 diffusion images with b = 1,000 *s*/*mm*^2^ and 5 T2-weighted b0 images. Each DTI slice was acquired with the following imaging parameters: 256 × 256 pixels per slice, 1.37 × 1.37 every pixel size, slice thickness = 2.70 *mm*, and flip angle=90°. More information about the scan parameters of images can be searched on the ADNI website (http://adni.loni.usc.edu/).

### 2.2. Preprocessing

All downloaded data have been preprocessed through a series of standard preprocessing procedures. Using the FMRIB software library (FSL) and FreeSurfer software, the T1-weighted sMRI images were preprocessed with skull-stripping, intensity normalization, and registration with a standard template Colin27 having the same coordinate system as MNI152. For DTI data, FSL was used to perform the preprocessing steps, including skull-stripping, eddy current correction, head motion correction, diffusion tensors estimation generating FA and MD maps, and registration with Colin27. Consequently, for each subject, we acquired three preprocessed and aligned 3D images in NIfTI file format from ADNI, which are sMRI, FA-DTI, and MD-DTI respectively. Three images have the same resolution of 110 × 110 × 110 voxels.

**Figure 1:**
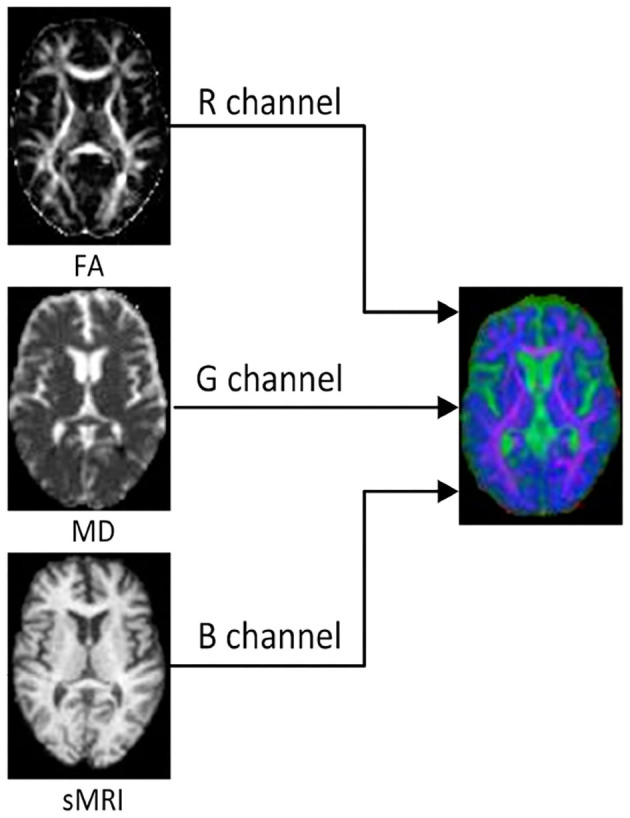
The FA, MD, and sMRI slice with the same index on the left are merged into an RGB slice on the right.

In order to reduce the interference of useless information and improve the computational efficiency, we investigated 2D slices of the above three kinds of images. For each subject, 32 slices with indexes 37 to 68 were selected from each kind of image. Then, the multi-modality fusion strategy were adopted: the slices with same index were merged into an RGB slice and saved as a JPEG image format, as shown in Figure 1. Consequently, each subject has 32 RGB slices which can be seen in Supplementary Material. It is worth noting that some researches have reported that the temporal lobe may make an essential contribution during the early stage of MCI (Bi et al., 2018; Cui et al., 2018; Zhang et al., 2019), and the 32 slices, including the whole temporal lobe and other memory related regions, such as the hippocampus and callosum, were thus selected.

All RGB slices were used to build the following two multi-modality dataset. A slice-level dataset consisting of 2,240 (32 × 70) EMCI RGB slices and 1,600 (32 × 50) NC RGB slices was built for training CNN model. In addition, the slice-level dataset was split into train set and validation set with the ratio of 8:2. Another subject-level dataset was built for SVM classification, which consists of 120 folders, each of which contained all RGB slices of one subject.

### 2.3. Proposed System Framework

**Figure 2:**
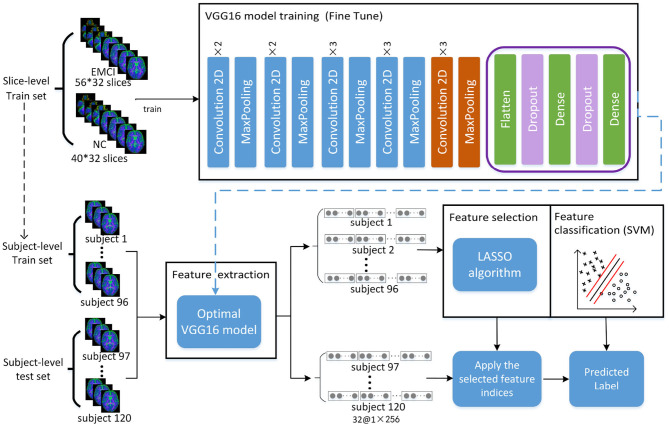
Total framework of proposed method, where the VGG16 model consists of five convolution blocks and a fully connected layer. For one slice, the output of VGG16 model is a matrix of 1 × 256. For each subject with 32 slices, 32 feature matrixes of 1 × 256 are acquired from VGG16, and they are then concatenated as a feature representation with the dimension of 1 × 8192. A total of 120 feature representations of 120 subjects are finally obtained, where 96 subjects used for training VGG16 network make up training set for feature selection and classification and 24 subjects make up test set for prediction.

The framework of the proposed approach is shown in Figure 2, where the VGG16 (Simonyan and Zisserman, 2014) network structure is exploited to perform feature extraction and an SVM classifier is used for feature classification. The dataset is split into five-folds, where four-folds (96 subjects) are used for training and one-fold (24 subjects) for testing. Firstly, the VGG16 network is trained with a slice-level training set using a transfer learning technique, and the optimal VGG16 model is then saved in terms of the lowest loss value. Secondly, all the slice features of each subject in the subject-level training set are extracted by the optimal VGG16 model. Thirdly, feature selection is performed by a LASSO algorithm to reduce the feature dimension and redundant information of training set, and then the outputs are used to train the SVM classifier to distinguish EMCI from NC. Finally, the features of the subject-level test set are extracted by optimal VGG16 model trained by training set, and the extracted features are further selected by LAASO model fitted by training set. After feature selection, the predicted labels are acquired through SVM classification.

#### 2.3.1. Convolutional Neural Network and Transfer Learning

The VGG16 network used for feature extraction in this study is one of the classical CNN. With the rapid development of deep learning, the CNN has achieved great success in large-scale image recognition in recent years. The CNN is mainly composed of a convolution layer, pooling layer, and fully connected layer (Yue et al., 2018). The convolution calculation is performed in the convolution layer with some convolution kernels, which can learn various features of the input images. The pooling layer can reduce the feature dimension and reduce vast network parameters and training time. The fully connected layer converges all learned features to produce the classification score of input data. In this study, due to the small dataset, transfer learning technique was used to train the VGG16 network to realize adaptation from source domain to target domain, which is to utilize the pre-trained weights to initialize the network whose structure is the same as pre-trained model trained with a larger dataset, and this has been applied to medical image classification in many studies (Pan and Qiang, 2010; Tajbakhsh et al., 2016; Hon and Khan, 2017; Karri et al., 2017; Kermany et al., 2018).

In this study, the pre-trained weights trained by nature image dataset Imagenet with 1,000 categories were transferred into VGG16 network. Due to the difference of categories number and image attributes, a fine-tuning strategy was taken. Firstly, the pre-trained weights of the first four convolution blocks were frozen, the fully connected layer was replaced, and then the pre-trained weights in the fifth convolution block shown in the orange part of Figure 2 and the initial weights of the new fully connected layer were continually updated until the model converged.

#### 2.3.2. LASSO

The feature matrix extracted from the VGG16 model possibly contains a lot of irrelevant or redundant features for EMCI diagnosis. To remove these features and reduce feature dimension, the feature selection algorithm of least absolute shrinkage and selection operator (LASSO) was adopted to select a small set of crucial features related to EMCI disease. LASSO is performed through minimizing the penalized objective function with L1 regularization which tends to give zero weight to irrelevant features so that the important features can be saved (Jiao et al., 2019). The objective function of LASSO is defined as follows:

(1) $$\begin{array}{r} f\left(\theta \right)=\frac{1}{2}||Y-X^{T}\theta |{|}_{2}^{2}+\lambda ||\theta |{|}_{1} \end{array}$$

where $X=\left[x_{1},x_{2},\ldots ,x_{N}\right]\in R^{d\times N}$ is a feature matrix. *N* is the number of subjects and *d* is the number of features. $Y={\left\{y_{i}|y_{i}\in \left\{-1,+1\right\}\right\}}_{i=1}^{N}$ is a set of corresponding class labels of subjects. θ represents a regression coefficient and λ is the regularization parameter to balance the complexity of the model.

#### 2.3.3. SVM

Support vector machine is most suitable classifier to deal with high-dimensional small dataset, which seeks a maximum margin hyper-plane to separate EMCI from NC. Given a training set ${\left\{x_{k},y_{k}\right\}}_{k=1}^{N}$ with input data $x_{k}\in R^{n}$ and corresponding binary class labels *y*_*k*_∈{−1, +1}, the output of primal SVM is presented as follows:

(2) $$\begin{array}{r} y\left(x\right)=sign\left[w^{T}\varphi \left(x\right)+b\right] \end{array}$$

Here, φ(*x*) is a non-linear function, mapping the input space to higher dimensional feature space, which makes the input data linearly separable in the hyperplane. *b* is a bias term. The optimization objective function is defined as follows (Suykens, 2001):

(3) $$\begin{array}{r} \underset{w,b,\xi }{min}J(w,\xi )=\frac{1}{2}w^{T}w+c\sum_{k=1}^{N}\xi_{k} \end{array}$$

subject to:

(4) $$\begin{array}{r} y_{k}\left[w^{T}\varphi \left(x_{k}\right)+b\right]\geq 1-\xi_{k},k=1,\ldots ,N,\xi_{k}\geq 0 \end{array}$$

ξ_*k*_ is a slack variable, indicating the tolerance of misclassification. *w* is the weight applied for input data *x*. *c* is a tuning parameter which must be a positive real constant.

### 2.4. Implementation

VGG16 network is trained based on Keras with a single GPU (i.e., NVIDIA GTX TITAN 12GB). The network is optimized by Root Mean Square Propagation (RMSProp) with a learning rate of 10^−4^. The weights update is performed in mini-batches of 32 samples per batch and stops after 50 epochs.

## 3. Results

### 3.1. Experiment Setup

In this study, several experiments are designed to validate the effectiveness of the proposed method in this paper. Specifically, we want to know whether multi-modality diagnosis can effectively improve classification performance than single modality. Firstly, slice-level dataset and subject-level datasets are built for FA, MD, and sMRI data. Except for modality fusion, these three single-modality datasets are built in the same way as a multi-modality dataset, as described in section 2. Then, the above three datasets and multi-modality dataset are used for EMCI diagnosis using the proposed method. Finally, the performance evaluation results of multi-modality are compared with that of single modality.

### 3.2. Feature Extraction

**Figure 3:**
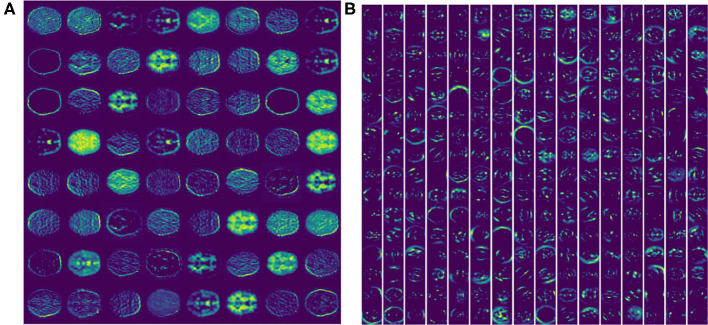
The feature activation maps of an RGB slice in the VGG16 model were visualized. **(A)** A total of 64 feature maps of 55 × 55 pixels from the first Maxpooling layer. **(B)** A total of 256 feature maps of 27 × 27 pixels from the seventh CONV layer.

Feature extracted from VGG16 model is abstract in deepest layer, but we can visualize the output of the lower layers. Figure 3A shows the output of the first *Maxpooling* layer of VGG16, where different filters (or convolution kernels) learn different features from various aspects. Some filters learn the brain shape, and others learn the interior structure of the brain. In deeper layers, such as the seventh convolution (CONV), the features become more and more localized, as shown in Figure 3B. It illustrates that although brain image is very different from nature image, the first few frozen layers can extract many generic features, such as side, angle, color, etc. In addition, the deeper fine-tuned layer can extract high-level target-specific features used for distinguishing different categories of images.

### 3.3. Feature Selection

**Figure 4:**
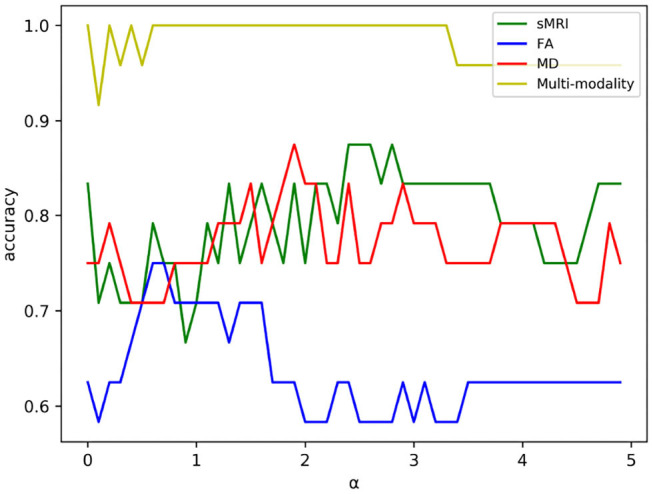
The variation curve of classification accuracy changing with α. Some values of α will arouse the problem of overfitting in a classification task, and such values were discarded in experiment.

Feature selection can reduce the effect of redundant features through adjusting the regularization parameter α. For one of the experiments in five-fold cross validation, the variation curve of classification accuracy changing with α is shown in Figure 4. As we can see, the value of α will affect the accuracy to some extent because the complexity of the model and the quantity of selected features rely on the value of α.

**Figure 5:**
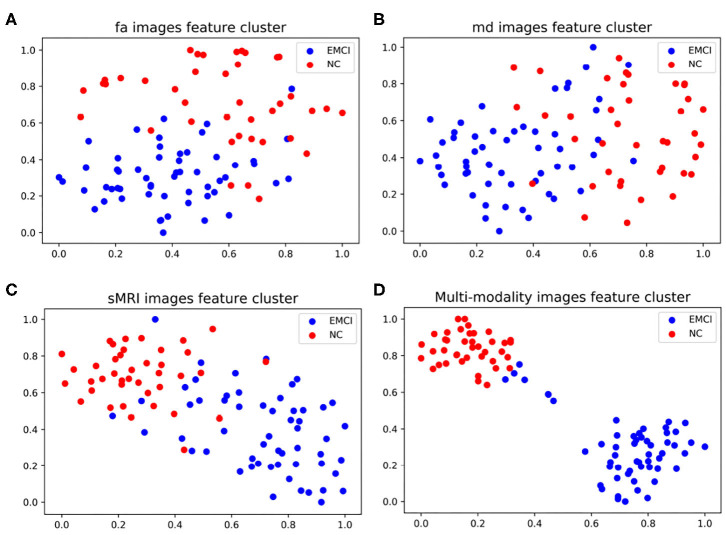
Manifold visualization of different neuroimaging feature, by t-SNE projection (Laurens and Hinton, 2008). **(A)** FA features; **(B)** MD features; **(C)** sMRI features; and **(D)** the features fusing FA, MD, and sMRI.

As shown in Figure 4, the classification accuracy of multi-modality data improves significantly comparing with single modality. In order to further validate the effectiveness of multi-modality data, the selected features are visualized in form of cluster figures, as shown in Figure 5. Single modality data can roughly distinguish EMCI from NC, especially sMRI data. The divisibility of multi-modality data is much better than that of any single modality data. It is worth noting that as shown in Figure 5D, the features of several EMCI subjects are similar to those of NC. The possible reason is that the differences between these EMCI subjects and NC subjects are especially subtle.

### 3.4. Feature Classification and Performance Evaluation

Before feature classification, data distribution of the selected features was linearly transformed to a normal distribution with unit standard deviation and zero mean. Then, the normalized features were fed into an optimal SVM classifier selected through exhaustive search. In order to alleviate the problem of data imbalance, class weights were imposed during SVM classification. The test accuracy (ACC), sensitivity (SEN), specificity (SPE), and area under the receiver operating characteristic curve (AUC) were finally acquired with five-fold cross-validation. We took the mean of each metric to evaluate classification performance quantitatively, and the classification results comparison between single modality and multi-modality method are shown in Table 2.

**Table 2:** Classification performance comparison between the single-modality and multi-modality method for distinguishing EMCI from NC.

**Method**	**ACC(%)**	**SEN(%)**	**SPE(%)**	**AUC(%)**
FA-DTI	67.5	71.4	75.7	68.6
MD-DTI	71.7	77.3	71.4	72.6
sMRI	73.3	78.8	77.1	73.9
FA+MD+sMRI	94.2	97.3	92.9	95.3

As shown in Table 2, the classification performance of multi-modality method is superior to that of single modality method. An average classification accuracy of 94.2%, a sensitivity of 97.3%, a specificity of 92.9%, and an AUC of 95.3% have been achieved to distinguish EMCI from NC with the multi-modality method, while the classification accuracy of FA, MD, and MRI data were 67.5, 71.7, and 73.3%, respectively. It is obvious that the method of combining the features from FA, MD, and sMRI data can enhance the classification accuracy significantly. These results indicate that it is difficult for single modality to represent all the attributes of EMCI because different modalities reflect pathological changes in different forms. For example, sMRI modality can show the change of macrostructure of brain while DTI modality can reflect the abnormity of microstructure of brain through random motion of water molecules affected by sensitive gradient field. The results also illustrate that DTI data is useful for distinguishing EMCI and NC to some extent. What's more, sMRI combined with DTI data can capture more disparate differences between EMCI and NC, which can improve classification performance greatly. This suggests that it is hihgly necessary to fuse data from different modalities for neuroimaging analysis.

### 3.5. Comparison With Other Methods

To our best knowledge, we are the first to fuse sMRI modality and DTI modality for distinguishing EMCI from NC. Table 3 provides an assessment of the proposed approach in comparison to related studies using the same metrics, where it can be clearly seen that the proposed method yielded the best results in all metrics. It is worth noting that the neuroimaging data of these competitive studies all comes from the ADNI website.

**Table 3:** Classification performance comparison with pervious researches for distinguishing EMCI from NC.

**Method**	**Modality**	**ACC(%)**	**SEN(%)**	**SPE(%)**
Prasad et al. (2015)	DWI	59.2	42.5	70.1
Forouzannezhad et al. (2018a)	sMRI	61.1	66.5	58.7
Raeper et al. (2018)	sMRI	81.0	83.3	78.57
Forouzannezhad et al. (2018b)	sMRI+PET	84.0	83.2	84.4
Proposed	sMRI	73.3	78.8	77.1
Proposed	sMRI+DTI	94.2	97.3	92.9

On the one hand, as described in section 1, paper (Forouzannezhad et al., 2018b; Raeper et al., 2018) both utilized a traditional machine learning method to classify EMCI and NC. We further introduced the CNN to perform feature extraction for multi-modality fusion data. As shown in Table 3, the proposed method using the sMRI modality alone outperformed paper (Forouzannezhad et al., 2018b), achieving an accuracy of 73.3%, 12.2% higher than paper (Forouzannezhad et al., 2018b), and the classification accuracy of using diffusion MRI alone shown in Table 2 is much higher than that of paper (Prasad et al., 2015), which shows the effectiveness of our method. It is worth noting that the preprocessing procedures of diffusion MRI in paper (Prasad et al., 2015) are the same as ours completely, which are performed by the group led by Pual M. Thompson. The above results illuminates that the CNN used in this study plays a significant role, which can efficiently extract different levels of features and reduce the error resulting from incomplete prior hypothesis. For example, despite the different pathology between EMCI and AD, many studies still assume that EMCI lesions are based on AD. Fortunately, CNN can ignore this difference by using full images instead of ROI as input and capture more features in a larger range. In other words, the CNN can extract the most discriminative features no matter what the pathology is. On the other hand, Raeper et al. acquired higher accuracy using sMRI data in paper (Raeper et al., 2018) than the proposed method; a possible reason is that transfer learning just can acquire a better performance when the target data are RGB images. We used the same three sMRI grayscale images to connect into a pseudo RGB image as the input of the CNN, and the relatively lower accuracy illustrates that the CNN utilizing transfer learning needs richer data to fit, such as the proposed multi-modality data.

Compared with the only method using multi-modality diagnosis, the proposed method using sMRI and DTI modality achieved an accuracy of 94.2%, which is 10.2% higher than that of paper (Forouzannezhad et al., 2018b), which used sMRI and PET modality. The right choice of multi-modality neuroimaging data and the introduction of CNN are the main reasons for acquiring high accuracy in the proposed multi-modality diagnosis. As we can see in Table 3, in the proposed method, the classification accuracy of using sMRI modality alone is 20.9% lower than that of fusing sMRI and DTI modality. It illustrates that DTI data can offer some extra complementary information for sMRI modality so as to improve classification performance significantly.

## 4. Discussion

Alzheimer's disease is an irreversible neurodegenerative disease, and identifying EMCI accurately contributes to early intervention of EMCI due to its high conversion rate to AD. However, studies of EMCI diagnosis suffer from some serious limitations, such as complex feature engineering and low accuracy. To solve these problems, we proposed a CNN-based multi-modality diagnosis method to distinguish EMCI from NC efficiently. Using the special multi-modality fusion method, we achieved a high classification accuracy of 94.2%, which is superior to other state-of-the-art methods. Three factors are identified as being potentially important. Firstly, DTI data is an effective supplement to sMRI, and it acts as an significant biomarker of EMCI since DTI data describes the changes of brain microstructure. Secondly, the transfer learning technique greatly improves the learning ability of small medical datasets and helps excavate more high-level target-domain features. Thirdly, it is very necessary to perform L1-norm feature selection by LASSO algorithm so as to remove redundant features in the high-dimensional features generating from multi-modality method. The contributions of different experimental steps were shown in Table 4. As we can see, LASSO and the transfer learning technique are both key steps and make almost an equal contribution to the proposed method.

**Table 4:** Classification performance comparison with different experiment setups, where *without LASSO* and *without fine-tuning*, representing transfer learning technique and LASSO algorithm, respectively, were not performed in the experiment.

**Method**	**ACC(%)**	**AUC(%)**
Without LASSO	90.0	92.8
Without fine-tuning	90.8	92.5
Without LASSO and fine-tuning	79.2	83.1
Complete experiment	94.2	95.3

*Complete experiment is the complete process of the proposed framework*.

In addition, compared with other multi-modality fusion methods (Cheng and Liu, 2017; Khvostikov et al., 2018), the training time and computing resources involved are reduced because the number of models is reduced using the proposed multi-modality fusion strategy. Other studies generally train multiple models to extract the features of different modalities and then fuse these extracted features. In the proposed method, only one model is trained for extracting the features of multi-modalities because the multi-modality data are input into the network through multiple channels.

In the past few years, several pioneering studies only focused on sMRI data to detect EMCI (Raeper et al., 2018; Yue et al., 2018; Taheri and Naima, 2019; Wee et al., 2019), and they have utilized morphological features and demographic factors to perform feature selection after the sMRI image is divided into 45 subcortical regions or 68 cortical regions. Interestingly, Wee et al. (2019) constructed cortical thickness graphs using sMRI data and input them into the popular graph CNN. sMRI is one of the common neuroimaging tool for disease diagnosis; however, there are many studies illustrating that multi-modality data are more effective than single-modality data for EMCI classification (Amoroso et al., 2018; Cheng et al., 2019; Forouzannezhad et al., 2020; Hao et al., 2020; Lei et al., 2020), and these studies have shown that different neuroimaging data may provide complementary information that is beneficial to diagnose EMCI. In addition, more and more researchers have turned their attention away from structural changes of the brain to functional change. They use fMRI data to construct a brain functional network of the EMCI and NC groups, and it has been found that the temporal lobe is the discriminating disease-related region. It is worth noting that in this study, we intentionally selected the 2D slices including the temporal lobe.

In this study, we applied transfer learning technique to train the CNN, which can alleviate the problems caused by a small dataset. Due to the small sample size of medical images, deep-learning-based diagnosis methods suffer many limitations. In paper (Puranik et al., 2018; Taheri and Naima, 2019), the classification accuracy is achieved by using 2D slices of neuroimaging data as input of the CNN, which is based on slice-level classification. In order to acquire the subject-level classification accuracy, we integrate all slice features of each subject.

In summary, multi-modality fusion diagnosis using the CNN is an effective medical image analysis method, and good classification performance can be achieved by selecting the most suitable neuroimaging modality according to the pathological characteristics of disease. Although it has been proven that DTI data is an effective imaging-biomarker for MCI diagnosis (Nowrangi et al., 2013; Marizzoni et al., 2016; Brueggen et al., 2017; Gyula, 2018), there are few studies to report. The high classification accuracy obtained in this paper again proves that DTI image can act as a remarkable biomarker for EMCI from the point of view of clinical diagnosis. The present study also posed several limitations. First, a larger sample size and other stages of cognitive impairment should be further considered to verify the stability and generalization ability of the proposed method. Secondly, the transfer learning technique used in this study relies on nature images. Although the fine-tuning method is adopted, a source domain model trained by brain images may fit the target domain better. These limitations will guide us to further enhance the robustness of the proposed method in future works.

## Data Availability Statement

Publicly available datasets were analyzed in this study. This data can be found here: http://adni.loni.usc.edu/.

## Author Contributions

All authors listed have made a substantial, direct and intellectual contribution to the work, and approved it for publication.

## Conflict of Interest

The authors declare that the research was conducted in the absence of any commercial or financial relationships that could be construed as a potential conflict of interest.
