# Supplementary material for: Identifying Early Mild Cognitive Impairment by Multi-Modality MRI-Based Deep Learning
Source: Front Aging Neurosci. 2020 Sep 4;12:206. doi: 10.3389/fnagi.2020.00206 (PMC7498722; doi:10.3389/fnagi.2020.00206)
Supplement: Supplementary file 1 [file Data_Sheet_1.docx]

Supplementary Material

# Supplementary Figures

##
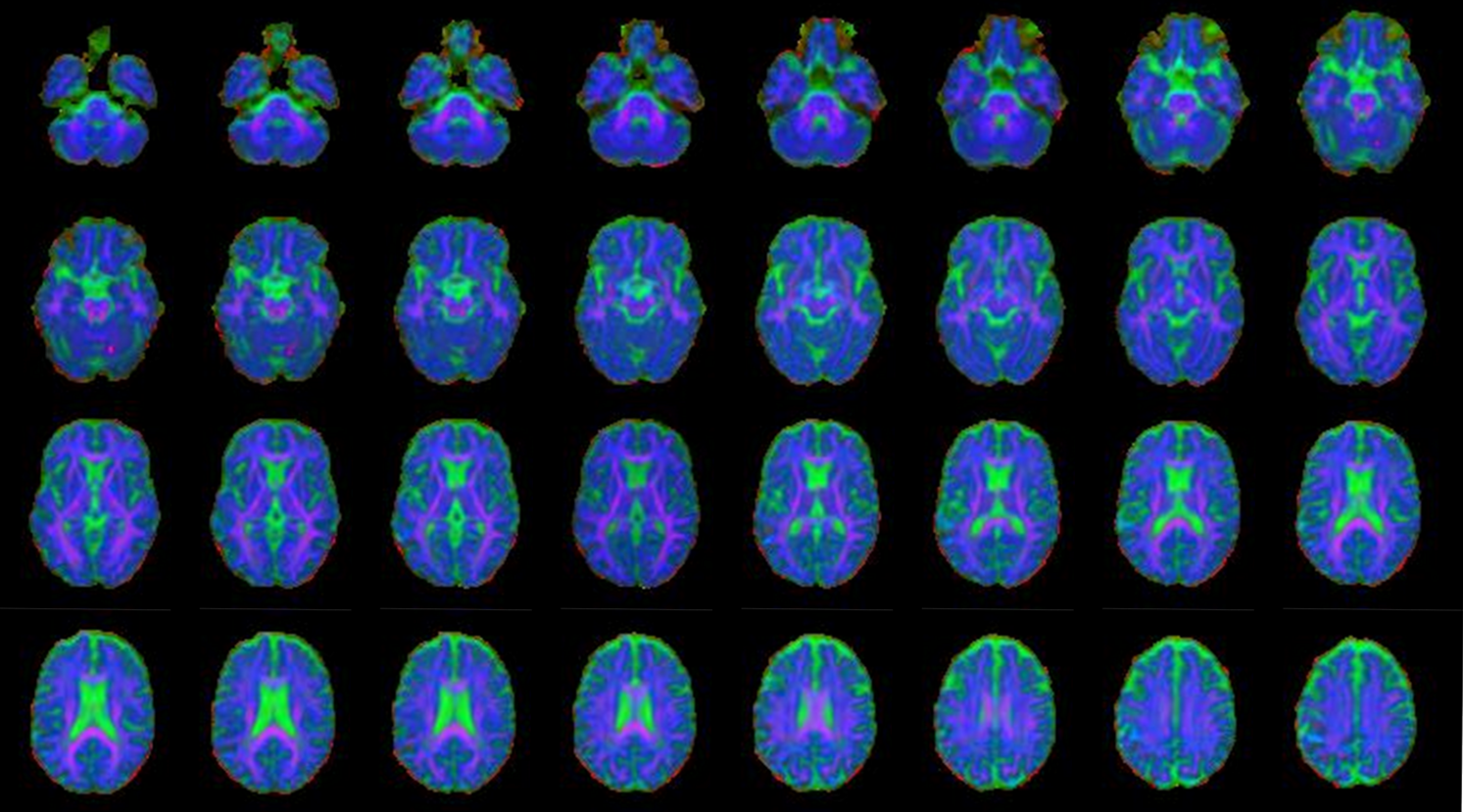
 Figures

**Figure S1.** 32 RGB slices from one subject with indexes ranging from 37 to 68.
